# Interfractional variation in whole-breast VMAT irradiation: a dosimetric study with complementary SGRT and CBCT patient setup

**DOI:** 10.1186/s13014-024-02418-5

**Published:** 2024-02-13

**Authors:** M. Mankinen, T. Virén, J. Seppälä, T. Koivumäki

**Affiliations:** 1 Deparment of Physics, University of Jyväskylä (JYU), Survontie 9 C, 40014 Jyväskylä, Finland; 2 Deparment of Medical Physics, Hospital Nova of Central Finland, Wellbeing Services County of Central Finland, Jyväskylä, Finland; 3 Center of Oncology, Kuopio University Hospital (KUH), The Wellbeing Services Country of North Savo, Kuopio, Finland

**Keywords:** Setup margin, Setup uncertainty, Tissue deformations, VMAT, CBCT

## Abstract

**Background:**

The dosimetric effect of setup uncertainty and tissue deformations in left-sided whole-breast irradiation with complementary surface-guided radiotherapy (SGRT) and cone-beam computed tomography (CBCT) setup was evaluated.

**Method:**

Treatment courses of 40.05 Gy prescribed dose in 15 fractions were simulated for 29 patients by calculating the dose on deformed CT images, that were based on daily CBCT images, and deforming and accumulating the dose onto the planning CT image. Variability in clinical target volume (CTV) position and shape was assessed as the 95% Hausdorff distance (HD95) between the planning CTV and deformed CTV structures. DVH metrics were evaluated between the planned and simulated cumulative dose distributions using two treatment techniques: tangential volumetric modulated arc therapy (tVMAT) and conventional 3D-conformal radiotherapy (3D-CRT).

**Results:**

Based on the HD95 values, the variations in CTV shape and position were enclosed by the 5 mm CTV-PTV margin in 85% of treatment fractions using complementary CBCT and SGRT setup. A residual error of 8.6 mm was observed between the initial SGRT setup and CBCT setup. The median CTV V95% coverage was 98.1% (range 93.1–99.8%) with tVMAT and 98.2% (range 84.5–99.7%) with 3D-CRT techniques with CBCT setup. With the initial SGRT-only setup, the corresponding coverages were 96.3% (range 92.6–99.4%) and 96.6% (range 84.2–99.4%), respectively. However, a considerable bias in vertical residual error between initial SGRT setup and CBCT setup was observed. Clinically relevant changes between the planned and cumulative doses to organs-at-risk (OARs) were not observed.

**Conclusions:**

The CTV-to-PTV margin should not be reduced below 5 mm even with daily CBCT setup. Both tVMAT and 3D-CRT techniques were robust in terms of dose coverage to the target and OARs. Based on the shifts between setup methods, CBCT setup is recommended as a complementary method with SGRT.

**Supplementary Information:**

The online version contains supplementary material available at 10.1186/s13014-024-02418-5.

## Background

Tissue deformations and positional variations during radiotherapy (RT) may compromise a carefully planned target coverage and deliver excess dose to critical organs-at-risk (OARs), such as lungs and the heart in the case of left-sided breast cancer (LSBC) treatments. A common way to account for uncertainties in patient setup and tissue deformations is to expand the clinical target volume (CTV) by a preset margin, forming the planning target volume (PTV). It is then assumed that the PTV to CTV marginal is sufficient to account for patient setup uncertainty, anatomical changes and treatment machine related uncertainties.

Advances in patient setup techniques have reduced the CTV-to-PTV margin [1] and varying estimates of the CTV-to-PTV margin in breast cancer therapy have been published. The Royal College of Radiologists has found a CTV-PTV margin of 5 mm suitable with modern image-guided radiotherapy (IGRT) systems [1], while some studies have proposed margins between 3 to 6 mm using kilovoltage radiograph (kV) or cone-beam computed tomography (CBCT) setup [2–6]. Commonly, van Herk [7] or Stroom [8] formulas have been used to define the uncertainty margin, even though the formulas do not account for uncertainties due to tissue deformations that may even outweigh the setup uncertainties [9].

Understanding the impact of changes in the target position and shape on the delivered dose is crucial. Advanced treatment techniques, such as the volumetric modulated arc therapy (VMAT), have been compared against more traditional techniques by means of simulation [4, 10–16]. Most of the previous studies have used single parameter approaches in order to quantify the impact of a single source of setup uncertainty, such as tissue swelling or translational shifts of the treatment isocenter. Realistic situations, however, include many possible combinations of positioning and tissue deformation related uncertainties that are difficult to quantify on their own.

This study evaluates the sufficiency of the preset 5 mm CTV-to-PTV margin with LSBC patients by assessing the positional and deformative variability of the CTV structure. The patients were positioned using daily CBCT imaging, that may be considered the gold standard in terms of setup accuracy [2, 17, 18]. While previous studies have evaluated the dosimetric impact of setup errors and deformations by simplified approaches [4, 10–16], this study evaluates the accumulated dose of the treatment course with the VMAT technique based on daily CBCT anatomy. As additional information, residual errors between the initial setup procedure with surface-guided RT (SGRT) system and CBCT setup are reported and compared with previous literature [3, 18–24], as the recent ESTRO guideline recommends complementary use of image-guided methods with SGRT [25].

## Methods

Thirty LSBC patients were retrospectively included in this study. The patients originally underwent a breast-conserving surgery and received whole-breast irradiation treatment in Kuopio University Hospital (KUH) between 2017 and 2021. The patients were imaged and treated in supine position. A breast board (C-Qual™ Breastboard, Civco Radiotherapy, USA) was used for positioning the arms above the head. The study protocol was approved by the research ethics committee of North Savo Hospital District.

The treatment planning CT (pCT) was acquired during deep-inspiration breath-hold (DIBH) using Siemens Somatom Definition AS Open 20 RT Pro edition with 512 × 512 image matrix using 3 mm slice thickness and approximately 1 mm pixel size. The patients were positioned on the CT table under free-breathing (FB) and a reference FB surface was acquired using the Sentinel system (C-RAD AB, Stockholm, Sweden). The SGRT system assessed the breath level using a region-of-interest placed on the sternum. The baseline level was set as the expiratory peak during relaxed breathing. Finally, a reproducible DIBH level was recorded with 3 mm tolerance and the patient was imaged during DIBH.

All the patients were prescribed a whole-breast irradiation dose 40.05 Gy in 15 fractions of 2.67 Gy [26]. CTV and OARs were contoured according to ESTRO guidelines [27]. The planning target volume (PTV) was created by expanding the CTV by 5 mm. The CTV and PTV were cropped by a 5 mm margin from the body surface contour, forming the CTVin and PTVin structures. The prescribed dose was normalized as the mean dose of the PTVin structure.

The tangential VMAT (tVMAT) planning approach was used for the clinical plans using 6 MV flattened beams [28]. Auto Flash tool (Elekta AB, Stockholm, Sweden) with a value of 20 mm was used to account for breast swelling and shape changes in the Monaco treatment planning system (Monaco v5.2, Elekta AB). The treatment planning goals for target coverage and OARs are presented in Table 1. Tangential 3D-conformal radiotherapy (3D-CRT) plans were retrospectively generated as a study reference in Eclipse treatment planning system (Varian Medical Systems Inc, Palo Alto, CA, USA). Field-in-field (FinF) technique was used with 2–4 subfields per each main field. Beams of 6 MV energy were used by default and accompanied by 15 MV beams in 3D-CRT planning to improve target coverage and avoid superficial hotspots when needed. The gantry angles were optimized by minimizing the beams-eye view (BEV) overlap with the heart and left anterior descending artery (LAD). The remaining heart and LAD volumes in BEV were shielded with multi-leaf collimator (MLC).

**Table 1:** Planning goals for tVMAT and 3D-CRT plans

Structure	Parameter	Goal
CTVin	V95%	> 98%
PTVin	V95%	> 95%
	V107%	< 1 cc
Heart	Mean	< 2 Gy
LAD	D1cc	< 16 Gy
Ipsilateral Lung	V16Gy	< 20%
	Mean	< 8 Gy
Contralateral Breast	Mean	< 1 Gy
Contralateral Lung	Mean	< 1 Gy
Ipsilateral humeral head	V15Gy	< 50%

The patients initially underwent treatment using Elekta Infinity linear accelerators equipped with Agility MLC and XVI system (Elekta AB) for CBCT acquisition. Two of these linacs were equipped with a Catalyst SGRT system (C-RAD AB), while one had a Catalyst HD SGRT system (C-RAD AB). Visual feedback was provided during CBCT and treatment fields in order to maintain the correct DIBH level. Catalyst c4D software (v. 6.1.2, C-RAD AB) was used to monitor patient surface position.

**Fig. 1:**
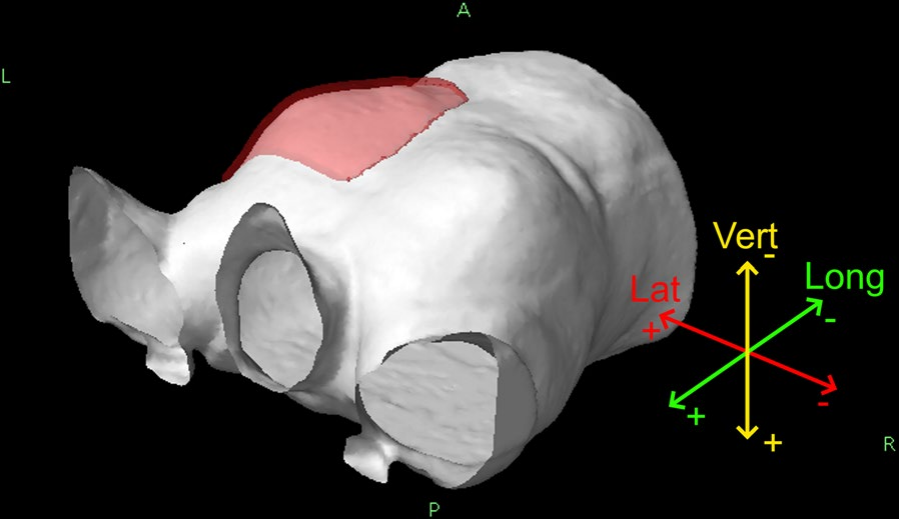
The lateral (Lat), longitudinal (Long) and vertical (Vert) principal axes with respect to the patient body. Left (L), right (R), anterior (A) and posterior (P) sides are denoted, and the left breast is highlighted by a red volume extending outside the body

At the start of each treatment fraction, the patients were positioned according to the reference FB surface, that was acquired before the pCT. The maximum allowed rotational deviance was ± 2 degrees in any direction. Automatic translations were applied to minimize the error between the reference FB surface and the live surface. This procedure is termed initial SGRT setup for the purposes of this study. The patient then performed DIBH for CBCT acquisition. During CBCT, the patient breathing level was monitored by using two circular reference points. The chest wall between the CBCT and the pCT was aligned first and additional corrections were added if breast swelling was observed. Since CBCT determined the final positioning, the combined SGRT and CBCT setup is termed CBCT setup in this study. Isocenter shifts between the initial SGRT setup to the CBCT setup positions were recorded, with directional choices presented in Fig. 1. Another reference DIBH surface was acquired after the DIBH for the first treatment field and used to monitor the patient’s postural stability for all the treatment fields within that fraction. The tolerance for automatic beam-off control was 5 mm for isocenter shift and 7 mm for any surface point.

**Fig. 2:**
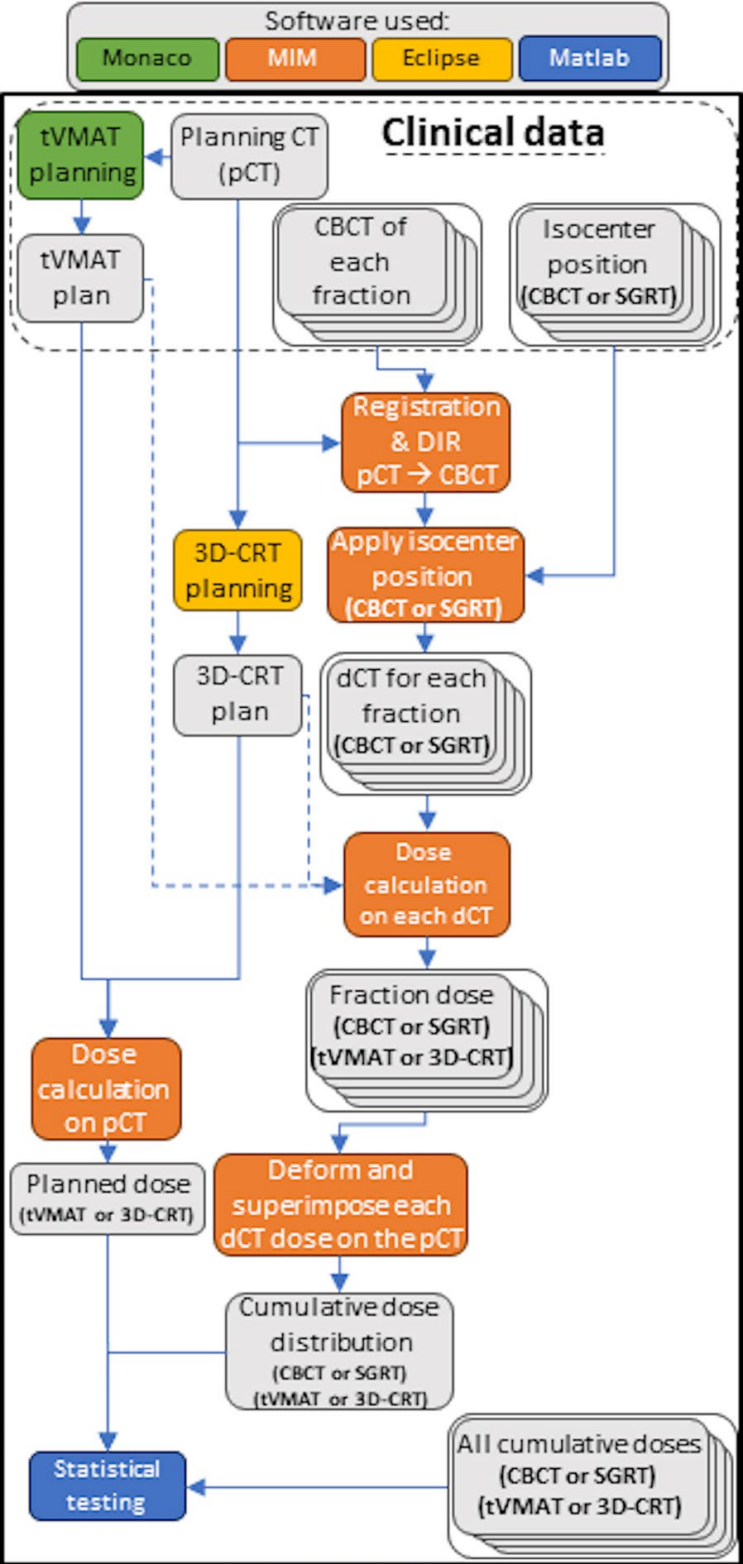
A flowchart representing the data processing. The legend for colors depicting the software used for each step is displayed at the top of the figure, while data is presented on gray background. (For interpretation of the references to color in this figure legend, the reader is referred to the web version of this article.)

Figure 2 presents a flowchart of image deformation and dose accumulation process. CT images representing each treatment fraction, henceforth called deformed CT images (dCT) in this study, were generated by deformable image registration (DIR) of pCT to CBCT using the Multimodality algorithm in MIM Maestro (MIM Maestro, MIM Software Inc., US). After the deformation, the dCT image position was adjusted in relation to the isocenter location during each fraction. The dCT represented the individual treatment fraction anatomy and setup. One patient was excluded from the analysis due to a missing CTV structure. The final patient cohort thus consisted of 29 patients and 435 treatment fractions.

Cumulative dose distributions were simulated by calculating the treatment fractions of 2.67 Gy on the dCT images and accumulating the dose on the pCT image by inverse DIR in MIM Maestro according to the tVMAT and 3D-CRT plans. Similarly, the dose accumulation with initial SGRT positioning was calculated. With the two treatment techniques and the two positioning methods, four different cumulative dose distributions were calculated. SciMoCa algorithm was used to calculate both planned and cumulative dose distributions in MIM Maestro software.

**Fig. 3:**
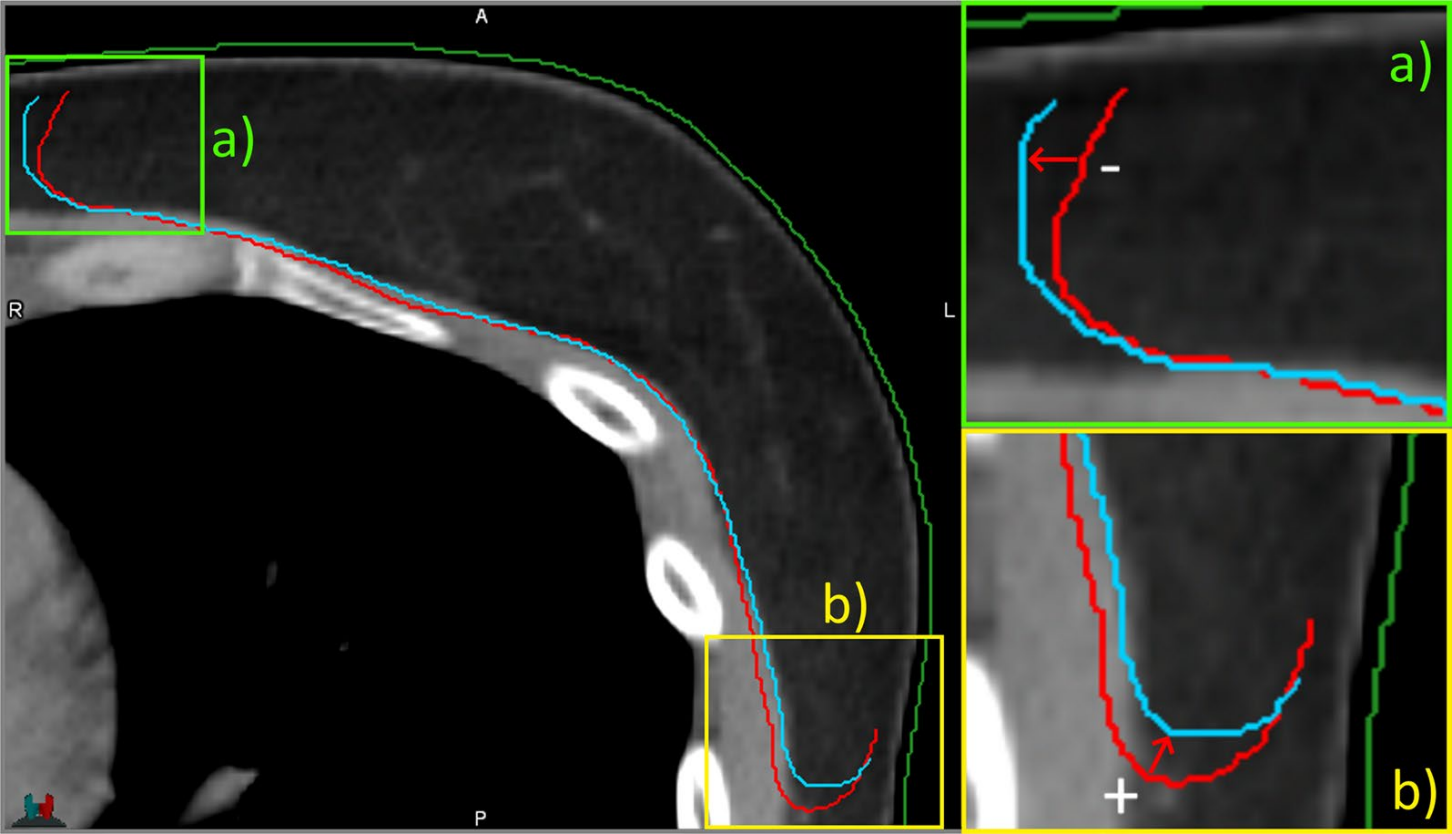
The choice of direction for pointwise distances from the dCT (red contour) to pCT (blue) CTVin contours. The cut images on the right demonstrate negative (**a**) and positive (**b**) distances from dCT CTVin to pCT CTVin. Thus, the positive HD95 value indicates the need for an additional margin, while the negative would indicate variations inside the original CTVin. The body contour of the pCT is depicted in dark green. (For interpretation of the references to color in this figure legend, the reader is referred to the web version of this article.)

To evaluate the variation in the shape of CTV dorsal and lateral edges and the position between dCT and pCT images, one-sided 95% Hausdorff distance (HD95) was calculated from the dCT CTVin contour to pCT CTVin contour. HD95 evaluates the 95% largest value of the set of shortest point-wise distances. New transversal planes were generated to the structure contour set with 1 mm interval by linear interpolation. New contour points were interpolated between the original contour points with 0.2 mm interval to reduce the error caused by uneven point spacing using an in-house developed Matlab script (v.2020b, MathWorks Inc, MA, USA). In order to assess the margin inside the body, all CTVin points within 5 mm of the pCT body surface were cropped. When calculating the one-sided HD95 from dCT CTVin to pCT CTVin, distances originating from inside the pCT CTVin were assigned as negative values (Fig. 3).

The dose-volume histogram (DVH) metrics of the cumulative dose distributions were evaluated against the planned values for tVMAT and 3D-CRT techniques. Dose homogeneity index (HI) was determined as 100 * (D2%—D98%)/D_pres_, where D2% and D98% correspond to the dose to the 2% and 98% volumes of the structure, and D_pres_ is the prescribed dose. Wilcoxon test was used to determine the statistically significant differences in DVH metrics (significance with *p* < 0.05). All statistical testing was executed in Matlab.

## Results

The median breast CTV volume was 950 cc (range 449–1466 cc). Breast edema was not observed in the CBCT images by visual inspection.

**Fig. 4:**
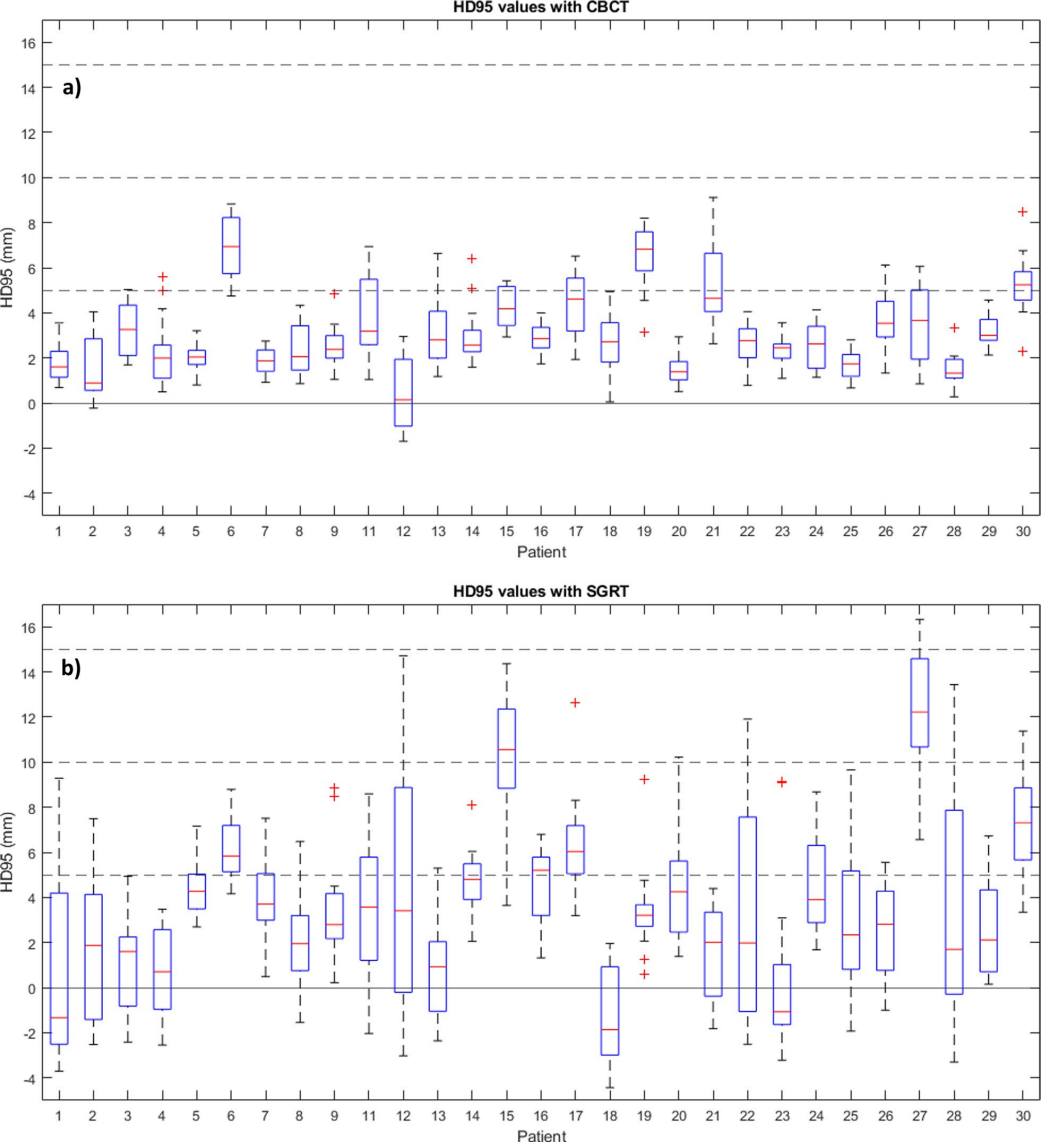
A box plot of HD95 values for the CTVin between dCT and pCT across all fractions for each patient (n = 29) with CBCT (**a**) and initial SGRT setup (**b**). The red line and the blue boxes indicate the median and 25th and 75th percentiles, respectively. The whiskers extend to the smallest and largest values within 1.5 times the interquartile range measured from the 25th and 75th percentiles, respectively. Outliers are marked with a plus sign. (For interpretation of the references to color in this figure legend, the reader is referred to the web version of this article.)

**Fig. 5:**
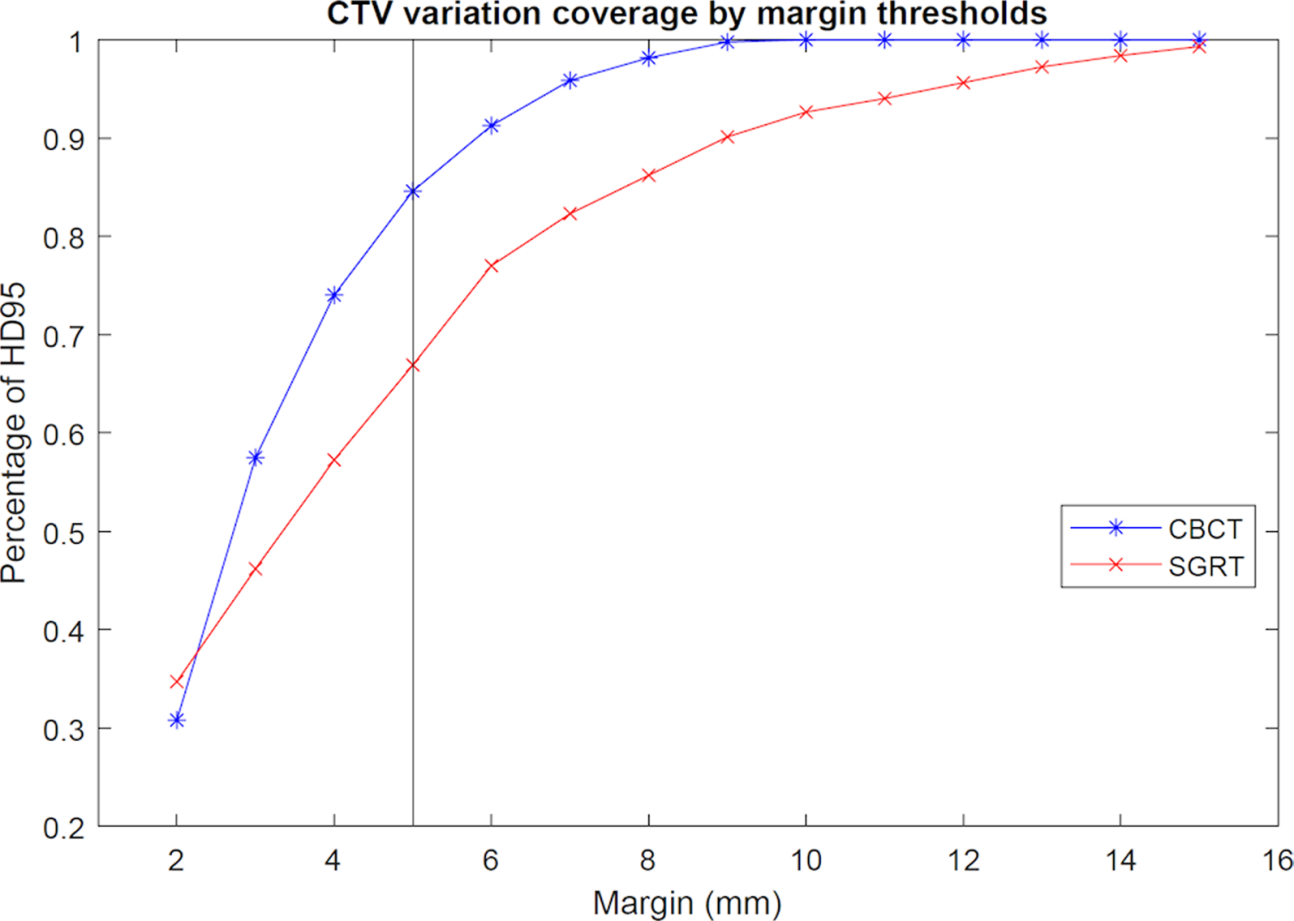
Percentage of treatment fractions where the variation of CTV outline exceeded the given margin thresholds. The variation was measured in HD95 for CBCT and initial SGRT setup methods. The vertical line marks the 5 mm margin that was used in planning. With CBCT, all the HD95 values were under 10 mm and only one fraction exceeded 9 mm

The median one-sided HD95 value across all fractions was 2.7 mm with CBCT setup and 3.4 mm with the initial SGRT setup (Table 2). Systematic deviation by a median of over 5 mm between dCT and pCT CTVin was observed in three patients (Fig. 4). HD95 values of under 5 mm were observed in 368 fractions out of the 425 total fractions, while a 10 mm margin would have been required to cover all the deformations across the treatment fractions (Fig. 5). 17 patients were observed to have their HD95 values below 5 mm across all fractions.

**Table 2:** The median one-sided HD95 values evaluated from dCT CTV to pCT CTV contour for CBCT and initial SGRT setups

Setup method	Median HD95 (mm)	Range (min–max)	Less than 5 mm
CBCT	2.7	− 1.7–9.1	368/425 (85%)
SGRT	3.4	− 4.4–16.3	291/425 (67%)

The range is indicated as minimum and maximum values. The third column indicates the number of fractions with HD95 values less than 5 mm

The initial treatment plans demonstrate the typical technique specific differences: higher target dose homogeneity and smaller high dose volumes for the heart and ipsilateral lung were achieved with tVMAT, while smaller low dose bath volume was achieved 3D-CRT (Table 3). All the planning goals were achieved in 11 initial plans with both techniques. As the CBCT field-of-view did not fully contain the OAR structures, only dose to 1 cc volume values (D1cc) are presented. Complete DVH data of the planned and cumulative dose distributions are presented in the Additional file 1: Tables S1–S6.

**Table 3:** Median DVH parameters of the planned and cumulative dose distributions with CBCT and initial SGRT setup

Structure		tVMAT Plan	tVMAT CBCT	tVMAT SGRT	3D-CRT Plan	3D-CRT CBCT	3D-CRT SGRT
CTVin	V95% [%]	98.5 (96.5–99.7)	98.1 (93.1–99.8)	96.3 * (92.6–99.4)	98.7 (95.7–99.7)	98.2 * (84.5–99.7)	96.6 * (84.2–99.4)
	HI	7.4 (5.0–9.9)	7.5 (5.0–12.5)	10.0 * (6.4–14.8)	9.9 (7.4–12.5)	9.9 (7.4–12.5)	10.0 * (7.4–14.9)
PTVin	V95% [%]	96.0 (94.3–98.9)	94.7 * (86.9–97.6)	89.9 * (80.0–95.1)	96.5 (92.2–98.2)	95.3 * (79.1–97.7)	89.8 * (79.3–95.9)
	D1cc [Gy]	42.0 (41.6–42.7)	41.7 * (40.9–42.7)	42.0 (40.8–42.8)	42.9 (42.2–43.5)	42.7 * (41.7–43.5)	42.7 * (41.8–44.1)
	HI	9.9 (7.1–12.4)	10.0 * (6.5–15.0)	24.7 * (8.3–44.6)	10.0 (9.5–36.8)	11.0 * (9.9- 22.3)	17.2 * (10.0–57.6)
Heart	D1cc [Gy]	5.8 (2.5–22.3)	6.0 (2.2–22.7)	4.2 * (2.4–19.4)	5.7 (1.7–15.9)	5.9 (1.4–18.7)	4.2 * (1.9–15.9)
LAD	D1cc [Gy]	7.8 (2.5–20.4)	7.7 (2.1–20.7)	5.7 * (2.8–17.3)	8.6 (1.6–26.3)	7.5 (1.3–21.2)	6.5 * (1.8–13.7)
Ipsil. Lung	D1cc [Gy]	38.3 (34.7–40.7)	37.5 * (35.1–40.1)	36.3 * (31.3–39.4)	39.3 (37.4–40.4)	38.8 * (37.5–40.2)	37.7 * (36.1–40.1)
Contral. Lung	D1cc [Gy]	2.1 (1.1–8.8)	2.2 (1.2–9.2)	2.0 * (1.1–8.9)	1.4 (0.7–3.3)	1.4 (0.7–3.3)	1.3 * (0.7–2.7)
Contral. Breast	D1cc [Gy]	4.7 (1.6–18.0)	4.4 (1.4–17.4)	3.3 * (1.3–14.2)	2.2 (1.2–37.7)	2.1 * (1.1–33.9)	1.7 * (0.9–23.8)
Ipsil. Hum	D1cc [Gy]	10.5 (1.1–35.1)	11.8 (1.3–35.7)	10.2 (1.1–35.3)	14.3 (1.0–38.7)	14.3 (1.4–40.9)	12.3 (1.0–39.1)

The range is indicated as minimum and maximum values. CBCT and SGRT columns mark the complementary SGRT/CBCT and initial SGRT setups, respectively. Statistically significant (p < 0.05) differences between planned and cumulative dose distributions are denoted with an asterisk (*)

**Fig. 6:**
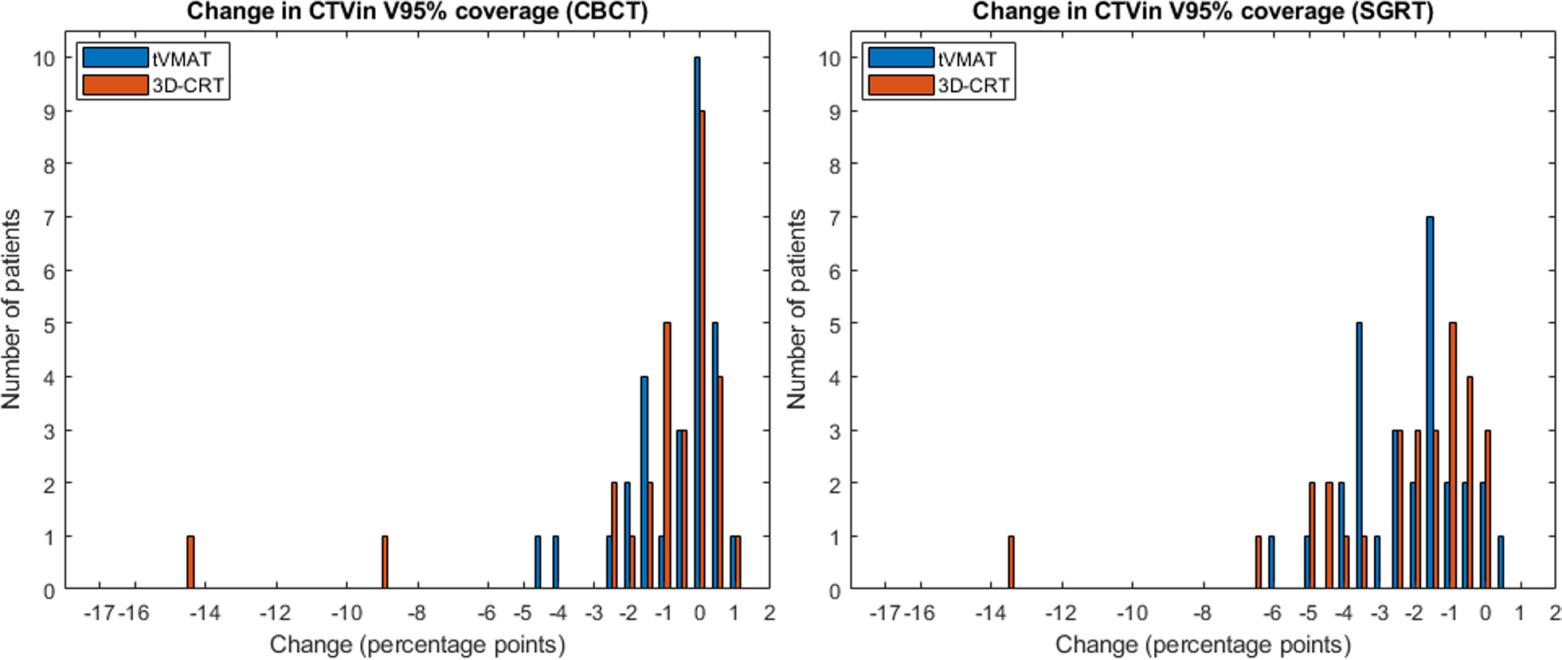
Histograms presenting differences in CTVin coverage with tVMAT (blue) and 3D-CRT (orange) with CBCT (left) and initial SGRT setup (right). Bin width of 0.5 percentage points was used

With CBCT setup, the cumulative dose distributions fulfilled all the DVH planning goals in 10 and 6 patients with tVMAT and 3D-CRT, respectively. The median CTVin coverage remained above the desired planning value, although a statistically significant decrease was observed for 3D-CRT (*p* < 0.05). During the planning stage, 66% of tVMAT plans and 86% of 3D-CRT plans fulfilled the desired goal for CTV coverage. In the cumulative dose distributions, 59% and 62% of patients retained the goal for CTV coverage with tVMAT and 3D-CRT, respectively. The PTVin HI increased with both techniques (*p* < 0.05), and more notably with 3D-CRT. Similar performance between the techniques is observed in the histograms of differences in CTVin and PTVin coverages (Fig. 6).

Statistically significant decreases in D1cc values were observed for ipsilateral lung with both techniques and for the contralateral breast with 3D-CRT (Table 3). Other OAR D1cc values were not significantly affected, however some large individual increases were observed with both planning techniques (Additional file 1: Tables S3 and S4).

Some outliers in the change in target coverage were found. Additional file 2: Fig. S1 presents an extreme outlier in target dose coverage with the 3D-CRT technique where the CTVin coverage changed by − 14.3 percentage points possibly due to body rotations and variation in the arm positioning with CBCT setup. In this case, the impact on tVMAT CTV coverage was only − 1.9 percentage points. Interestingly, the coverage only changed by − 6.3 percentage points with SGRT setup. Conversely, two other patients demonstrated differences of − 4.8 and − 3.8 percentage points in CTVin coverage with tVMAT, whereas 3D-CRT coverage changed by − 1.7 and 0.3 percentage points with CBCT setup. Variation in the lateral adipose tissue was observed during the fractions, but no single cause for the decline in coverage could be determined in either case. The former tVMAT outlier is presented in Additional file 2: Fig. S2.

With the initial SGRT setup, only 1 and 2 plans retained the planning goals with tVMAT and 3D-CRT, respectively. The DVH parameters with the initial SGRT setup demonstrate substantially weaker dosimetric results compared to CBCT setup. A considerable systematic median vertical offset of 6.2 mm (range: − 9.9–16.0 mm) in the posterior direction was observed between initial SGRT and CBCT setup (Table 4). The corresponding lateral and longitudinal shifts were 0.2 mm (range: − 9.2–12.3 mm) and 0.5 mm (range: − 11.4–15.0 mm). The median residual error between the initial SGRT and CBCT setup was 8.6 mm (95% percentile 13.6 mm, range between 1.0 and 21.7 mm).

**Table 4:** The isocenter shifts along the principal axes from initial SGRT position to final CBCT setup position

	Median (mm)	Min / Max (mm)	Median magnitude (mm)	95% percentile (mm)
Lat	0.2	− 9.2/12.3	2.0	7.0
Long	0.5	− 11.4/15.0	2.8	9.9
Vert	6.2	− 9.9/16.0	6.5	11.3

Lat, Long and Vert denote the lateral, longitudinal and vertical axes

## Discussion

This study evaluated the sufficiency of the 5 mm CTV-to-PTV margin and the dosimetric effects in the presence of setup uncertainties and tissue deformations in breast cancer RT. The 5 mm margin was sufficient to account for CTV deformations only in 85% of the treatment fractions using daily CBCT setup. In the cumulative dose distributions with CBCT setup, the goal for CTV dose coverage was fulfilled in 59% and 62% of patients with tVMAT and 3D-CRT, respectively. Therefore, reducing the margin below 5 mm is unadvisable. The results also argue for caution in patient positioning, as less accurate methods may further compromise the target dose coverage.

While the CTV-to-PTV margin can be reduced to 5 mm with modern IGRT techniques [1], this study showed that a 5 mm margin is not enough to enclose all anatomical and setup uncertainties of the CTV inside the body. CTV-to-PTV margins of 7 and 10 mm during treatment planning would have enclosed 95% and 100% of CTV shape changes, respectively (Fig. 5). An increase in margin would probably improve the delivered dose coverage, but the effect of increasing the enclosure percentage on local recurrence probability is not known.

The cumulative dose distributions demonstrated a median decline of 0.4 and 0.5 percentage points in CTVin coverage with tVMAT and 3D-CRT, respectively, while the median coverages remained above the planning goal with both techniques. Therefore, both techniques proved robust in this patient cohort. The dosimetric effects in the cumulative dose distributions were caused by both tissue deformation and setup uncertainties that were incorporated in the deformed CT images. The median CTV coverage values of cumulative dose distributions fulfilled the initial planning goal, even though the planning goal of V95% > 98% of prescribed dose was relatively strict. A larger impact was demonstrated in PTVin coverage, which was retained with 3D-CRT and slightly declined below the planning goal for tVMAT. This effect was to be expected as random position error mainly manifest as blurring on the edges of a homogeneous dose distribution [7]. The range of CTVin and PTVin coverages was larger for 3D-CRT compared to tVMAT, but the median values indicate that both techniques are robust towards positional and deformative uncertainties in this patient cohort.

Previous studies investigated dosimetric effects using more simplified methods. Van der Veen et al. utilized deformed CT images based on CBCT, demonstrating differences of -2% to + 0.5% in CTVin coverage [15]. Rossi et al. found a minor decline in PTVin coverage with CBCT-based tissue modifications [12]. Our study utilized a more comprehensive simulation method with CBCT images from each treatment fraction, as opposed to three to five CBCT images, and supports these findings [12, 15]. By contrast, Dekker et al. achieved CTVin V95% coverage above 98% in 90% of patients with tangential IMRT and hybrid IMRT techniques by calculating the dose on the CBCT images [16]. However, a higher proportion of their plans met initial planning goals compared to clinical tVMAT and 3D-CRT reference plans in this study. Similar to van der Veen et al. and Rossi et al., Dekker et al. included only three to four CBCT images.

Unlike this study, most of the dosimetric studies considering breast cancer treatment uncertainties have only incorporated one individual uncertainty without daily CBCT. Hennet et al. demonstrated a 4% decline in PTVin V95% coverage with 4 to 7 mm isotropic swelling by using a conventional VMAT technique without avoidance sectors [10]. Rossi et al. simulated worst-case scenarios with tVMAT and isotropic expansion of the breast of 4, 8 and 12 mm [11]. With a 20 mm Auto Flash setting, the initially acceptable chest wall CTV (commonly the CTVb/c) V95% coverage declined to 90% with 4 mm isotropic swelling [11]. However, the PTVin V95% coverage remained clinically acceptable. According to Rossi et al., swelling of the target breast up to 4 mm is to be expected roughly in 50% of the patients [12]. Likewise, Seppälä et al. reported breast expansion of less than 3 and 5 mm in 38% and 66% of patients during the radiotherapy course of the breast, respectively [29]. In this study, the dosimetric impact in the presence of anatomical deformations was small compared to the referred studies [10, 11].

A common method for assessing the dosimetric effect of setup uncertainty is to apply rigid translations and recalculate the dose on the planning CT image. Based on this method, Jensen et al. applied clinical online match shifts to the planning CT image [4]. Similar to this study, they observed a 1 Gy decline in PTV D95% and only a 0.1 Gy decline in CTV D98% with VMAT robust optimization [4]. Ding et al. and Zhao et al. concluded that the effects of 3 mm shifts are rather negligible, but larger shifts demonstrated greater impact with VMAT compared to hybrid IMRT [13] and 3D-CRT [14] techniques. In the present study, the tVMAT technique proved robust in the presence of realistic uncertainties in the CTV edge position inside the body, that were less than 5 mm in 85% of fractions. It is noteworthy, that some studies [13, 14] did not report the use of skin flash technique, even though applying a skin flash is recommended in breast cancer treatment planning [29, 30].

This study only reported D1cc values for OAR structures as they were only partially visible in the CBCT field-of-view, thus rendering the DIR unreliable for the unseen parts of the OARs. No statistically significant increases were demonstrated in D1cc of any OAR, indicating that both tVMAT and 3D-CRT techniques are robust in terms of dose to normal tissue. Some increases in D1cc were found using CBCT setup, as the maximum increase in D1cc of the heart and LAD was larger for 3D-CRT. On the other hand, the maximum increase in D1cc of the contralateral breast was larger with tVMAT (Additional file 1: Tables S1–S6).

The residual error between initial SGRT setup and CBCT setup observed in this study (median 8.6 mm, 95% below 13.6 mm) was large compared to other studies. The largest reported average residual errors were 6–7 mm in magnitude [20, 21]. Much smaller component-wise values of < 2 mm have also been reported [19, 24]. Similar to this study, average lateral and longitudinal directions (0.2 and 0.5 mm) were observed by Cravo Sá et al. [22]. They also observed a pronounced vertical shift of 2.1 mm. A consistent bias in the median vertical shift was observed (6.2 mm vs. < 1 mm in other directions) at the authors’ clinic despite routine quality assurance of the SGRT system. A potential cause for the bias is that the live and reference FB surface for initial SGRT setup corresponded to different respiratory phases at the moment the automatic translations were applied. Other potential causes for the bias are intra-fraction movement and inter-operator variability in SGRT setup. In addition, breast surface deformation has been shown to cause uncertainty in SGRT positioning [31]. Based on these results, IGRT verification of the patient position is recommended to avoid systematic set up errors.

The recent ESTRO-ACROP guideline for SGRT [25] recommends the use of a defined protocol and verification of SGRT-only positioning by IGRT at least weekly, if SGRT is used without daily IGRT. However, IGRT offers greater accuracy of patient positioning when compared to SGRT [3, 18, 20–23, 25]. The results for CTVin and PTVin dose coverages of the present study demonstrate that no further uncertainty should be added on top of CBCT setup. This further reinforces the need of IGRT methods in conjunction with the initial SGRT setup. Particularly when using the VMAT technique, CBCT setup has been recommended over 2D kV setup [12].

Some limitations exist in this study. The pCT to dCT deformation based on the CBCT image is prone to exaggerating the expansion of the body tissue into air, if the air-tissue interfaces are blurred in the CBCT image. Moreover, the uncertainties in the DIR accuracy may have affected the shape of the dCT CTV structures to some degree. Finally, intra-fraction motion effects, such as variability in DIBH stability and reproducibility across multiple breath holds [32], were not incorporated in the dose accumulation process.

While CBCT setup combined with the 5 mm CTV-to-PTV margin could not account for all setup and deformation related uncertainties, good practice aims to minimize the setup and dosimetric errors. For this purpose, this study proved that there is little difference between tVMAT and conventional 3D-CRT techniques in terms of median CTV coverage and dose to OARs. Looking forward in breast cancer radiotherapy, the implementation of ultra-hypofractionation [33] might allow for even less uncertainty in patient setup thus highlighting the value of CBCT even when SGRT systems are used. However, the assessment of dosimetric uncertainty with only five fractions remain a subject for future investigations.

## Conclusions

The CTV-to-PTV margin should not be reduced below 5 mm even with daily CBCT setup in whole-breast irradiations. Both tVMAT and 3D-CRT techniques were found clinically robust against setup and tissue deformation uncertainties. Complementary use of CBCT and SGRT in patient setup is recommended over SGRT-only setup.

### Supplementary Information

**Additional file 1**. **Table S1** The tVMAT planning DVH parameters for each patient. **Table S2** The 3D-CRT planning DVH parameters for each patient. **Table S3** The tVMAT DVH parameters of the cumulative dose for each patient with CBCT setup. **Table S4** The 3D-CRT DVH parameters of the cumulative dose for each patient with CBCT setup. **Table S5** The tVMAT DVH parameters of the cumulative dose for each patient with initial SGRT setup. **Table S6** The 3D-CRT DVH parameters of the cumulative dose for each patient with initial SGRT setup.

**Additional file 2**. **Fig. S1** A case example of the cumulative CTV dose coverage decline on the planning CT image with 3D-CRT. **Fig. S2** A case example of the cumulative CTV dose coverage decline on the planning CT with tVMAT.

## Data Availability

The datasets generated and analyzed during the current study are not publicly available due to protection of individual patient privacy but are available from the corresponding author on reasonable request and with permission of Wellbeing services county of North Savo.

## Abbreviations

2D kV: Two-dimensional kilovoltage
3D-CRT: Three-dimensional conformal radiotherapy
BEV: Beam’s eye view
CBCT: Cone-beam computed tomography
CT: Computed tomography
CTV: Clinical target volume
dCT: Deformed CT
DIBH: Deep-inspiration breath-hold
DIR: Deformable image registration
DVH: Dose-volume histogram
ESTRO: European society for radiotherapy and oncology
FinF: Field-in-field
HD95: 95% Hausdorff distance
HI: Homogeneity index
IGRT: Image-guided radiotherapy
IMRT: Intensity-modulated radiotherapy
LAD: Left anterior descending artery
Lat: Lateral
Long: Longitudinal
LSBC: Left-sided breast cancer
MLC: Multi-leaf collimator
MV: Megavoltage
OAR: Organ-at-risk
pCT: Planning CT
PTV: Planning target volume
RT: Radiotherapy
SGRT: Surface-guided radiotherapy
tVMAT: Tangential volumetric modulated arc therapy
Vert: Vertical
VMAT: Volumetric modulated arc therapy
