# Supplementary material for: Interfractional variation in whole-breast VMAT irradiation: a dosimetric study with complementary SGRT and CBCT patient setup
Source: Radiat Oncol. 2024 Feb 13;19:21. doi: 10.1186/s13014-024-02418-5 (PMC10863193; doi:10.1186/s13014-024-02418-5)
Supplement: Supplementary file 2 — Additional file 2. Fig. S1 A case example of the cumulative CTV dose coverage decline on the planning CT image with 3D-CRT. Fig. S2 A case example of the cumulative CTV dose coverage decline on the planning CT with tVMAT. [file 13014_2024_2418_MOESM2_ESM.pdf]

Interfractional variation in whole-breast VMAT irradiation – a dosimetric study with complementary SGRT and CBCT patient setup

Authors: Mankinen M, Virén T, Seppälä J, Koivumäki T

Supplementary Material

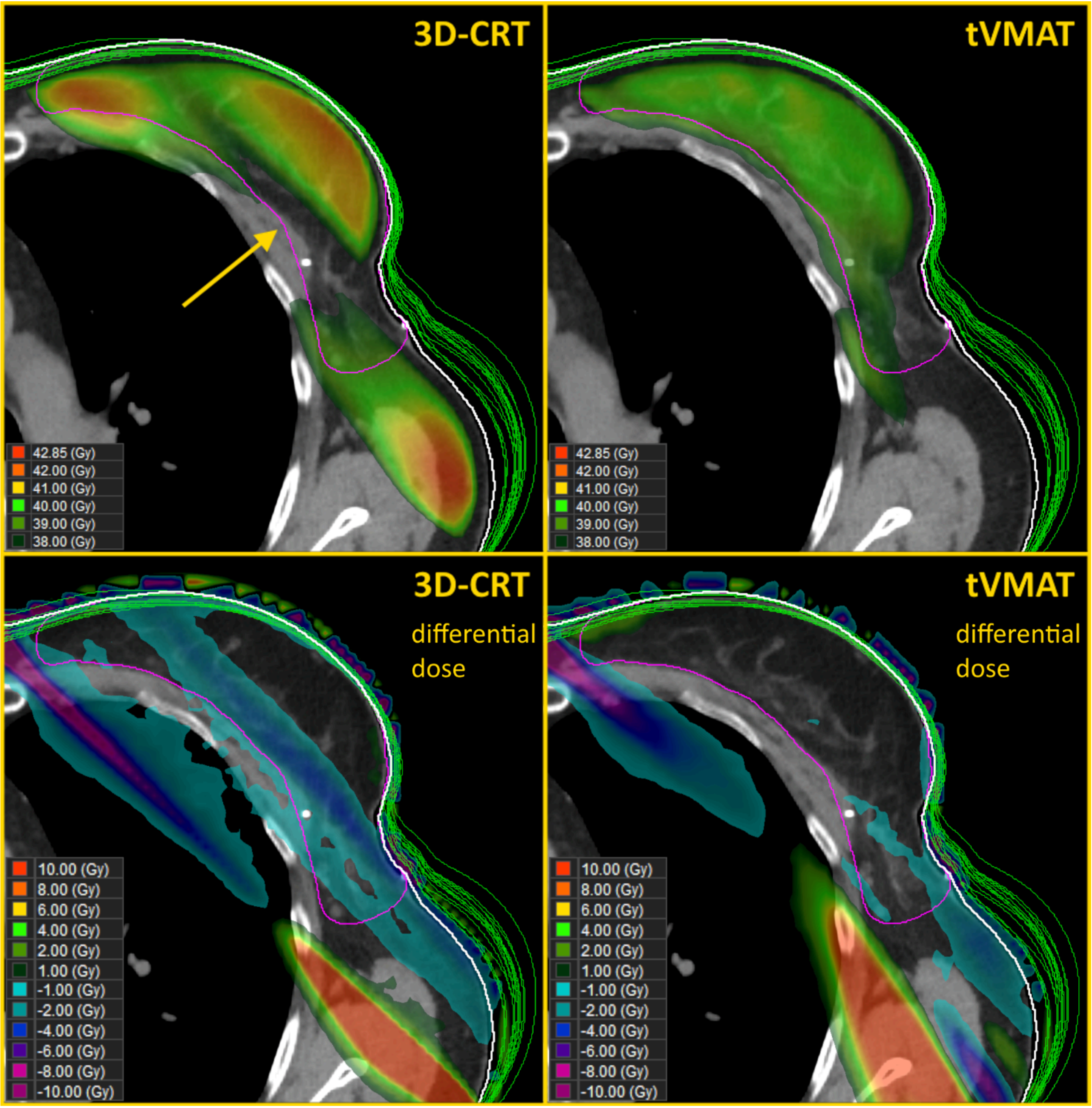

Supplementary Figure 1: A case example (Patient 11) presenting the perturbed dose after dose accumulation based on CBCT setup and the differential dose on planning CT image with 3D-CRT and tVMAT techniques. Differential dose was calculated as perturbed dose minus planned dose. The yellow arrow demonstrates a decline in dose coverage of the planning CTV (purple contour) with 3D-CRT technique. The cause for the decline in coverage is the variation in body outline positions (green outlines), that coincided with the posterior tangential field central axis during the treatment fractions. In this case, tVMAT dose demonstrates a smaller effect.

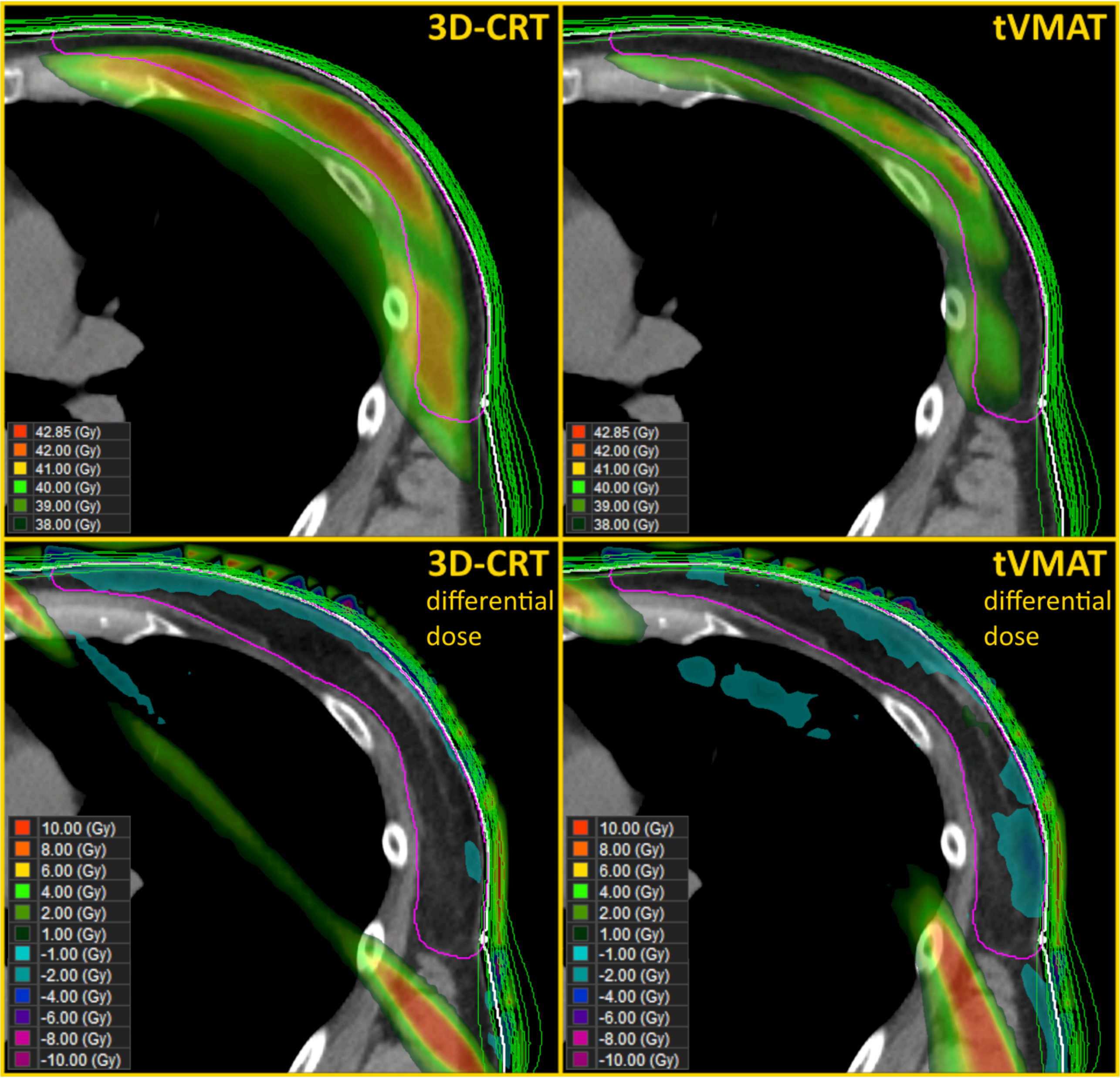

Supplementary Figure 2: A case example (Patient 28) presenting the perturbed dose after dose accumulation based on CBCT setup and the differential dose on planning CT image with 3D-CRT and tVMAT techniques. Differential dose was calculated as perturbed dose minus planned dose. The tVMAT dose distribution presents a decline in CTV (purple contour) coverage near the skin surface. The white outline represents the planning body outline while the green outlines represent the body outlines on different treatment fractions.
